# Efficacy Assessment of the Co-Administration of Vancomycin and Metronidazole in *Clostridioides difficile*-Infected Mice Based on Changes in Intestinal Ecology

**DOI:** 10.4014/jmb.2312.12034

**Published:** 2024-02-29

**Authors:** Saiwei Zhong, Jingpeng Yang, He Huang

**Affiliations:** 1 College of Biotechnology and Pharmaceutical Engineering, Nanjing Tech University, Nanjing 211816, P.R. China; 2 School of Food Science and Pharmaceutical Engineering, Nanjing Normal University, Nanjing 210023, P.R. China

**Keywords:** Vancomycin, metronidazole, *Clostridioides difficile* infection, co-administration, fecal microbiota, metabolome

## Abstract

Vancomycin (VAN) and metronidazole (MTR) remain the current drugs of choice for the treatment of non-severe *Clostridioides difficile* infection (CDI); however, while their co-administration has appeared in clinical treatment, the efficacy varies greatly and the mechanism is unknown. In this study, a CDI mouse model was constructed to evaluate the therapeutic effects of VAN and MTR alone or in combination. For a perspective on the intestinal ecology, 16S rRNA amplicon sequencing and non-targeted metabolomics techniques were used to investigate changes in the fecal microbiota and metabolome of mice under the co-administration treatment. As a result, the survival rate of mice under co-administration was not dramatically different compared to that of single antibiotics, and the former caused intestinal tissue hyperplasia and edema. Co-administration also significantly enhanced the activity of amino acid metabolic pathways represented by phenylalanine, arginine, proline, and histidine, decreased the level of deoxycholic acid (DCA), and downregulated the abundance of beneficial microbes, such as *Bifidobacterium* and *Akkermansia*. VAN plays a dominant role in microbiota regulation in co-administration. In addition, co-administration reduced or increased the relative abundance of antibiotic-sensitive bacteria, including beneficial and harmful microbes, without a difference. Taken together, there are some risks associated with the co-administration of VAN and MTR, and this combination mode should be used with caution in CDI treatment.

## Introduction

*Clostridioides difficile* is a gram-positive anaerobic bacterium capable of producing spores [1]. As a common, conditionally pathogenic bacterium in the intestinal tract, most *C. difficile* strains produce two pathogenic toxin proteins, A and B, in the presence of gut dysbiosis, which can synergistically destroy intestinal epithelial cells, thereby inducing intestinal inflammation and tissue damage, both of which are symptoms of *C. difficile* infection (CDI) [2]. The initial symptom of CDI is primarily diarrhea, or *C. difficile*-associated diarrhea (CDAD), which accounts for nearly 25% of all antibiotic-associated diarrhea (AAD) cases, making it a public health and economic cost concern worldwide [3]. Primary *C. difficile* infection (pCDI) is usually treated with traditional first-line antibiotics, including the use of vancomycin (VAN) and metronidazole (MTR), but is accompanied by a high rate of recurrence, and recurrent *C. difficile* infection (rCDI) is more difficult to cure. There are various reasons behind this phenomenon, including an imbalance of host immune homeostasis, the emergence of antibiotic-resistant strains, difficulty in killing *C. difficile* spores, and disruption of the intestinal ecological environment [4]. For the treatment of pCDI, VAN and MTR alone are equally effective, mainly in terms of mortality and recurrence rates, while for rCDI, VAN is significantly more effective than MTR [5]. There was no significant difference in clinical cure rates between MTR and VAN in the treatment of CDI in children and adolescents, but in a subgroup analysis, MTR was found to have significantly lower clinical cure rates than VAN in the U.S. and Europe [6]. Although the European Society of Clinical Microbiology and Infectious Diseases (ESCMID), the Society for Infectious Diseases of America, and the Society for Healthcare Epidemiology of America no longer recommend MTR as the standard of care for CDI, this dosing regimen is not absolute in different regions; for example, MTR is more appropriate for the treatment of pCDI in Korea [7-9]. However, dosing regimens that combine VAN and MTR for the treatment of CDI are infrequent, with some studies showing no significant difference in the efficacy of the combination compared to single antibiotics [10]. In fact, this co-administration model has not been fully evaluated, and its actual efficacy and potential risks are not known.

The gastrointestinal tract serves as the principal site for antibiotic activity and the establishment of *C. difficile* colonization and infection. Therefore, a deeper understanding of the changes in the host intestinal ecology can further reveal the differences in efficacy brought about by specific antibiotic treatments. Some studies have revealed the perturbation of microbiota and changes in microbial composition resulting from the use of VAN or MTR alone [11, 12]. Although previous study efforts have explored the perturbations caused by the VAN-MTR combination mode on the microbiota, these studies have centered on the CDI prophylaxis phase, *i.e.*, the early intake of antibiotics that disrupts the microbiota before examining susceptibility to *C. difficile* invasion [12-15]. For example, Lewis *et al*. found that oral VAN caused more pronounced disruption of the gut microbiota in mice than MTR, weakened gut resistance to colonization by *C. difficile*, and led to intensive colonization by VAN-resistant *Enterococci*, **Klebsiella* pneumoniae*, and *Escherichia coli* [12]. To our knowledge, little is known about the changes in the microbiota in the context of an established infection that is in the onset phase and requires antibiotic treatment.

Overall, the therapeutic effect of the co-administration of VAN and MTR on CDI and the changes in the intestinal ecological environment caused by *C. difficile*, especially the changes in the gut microbiota and metabolites, have not been thoroughly investigated. Therefore, in this study, we evaluated the therapeutic effects of VAN and MTR in single or combination mode on pCDI mice, focusing on the change patterns and characteristics of fecal microbiota and key metabolites under co-administration, to provide a reference basis for the combination of these two antibiotics.

## Materials and Methods

### Strains and Mice

*C. difficile* ATCC 43255 (VPI 10463, CD) was purchased from the American Type Culture Collection (ATCC, USA). This strain was inoculated in fresh brain heart infusion (BHI) broth and cultured in an anaerobic chamber (Mitsubishi, Japan) containing an anaerobic gas-producing bag (AnaeroGenTM, Oxoid Ltd., UK) at 37°C. A total of 70 specific pathogen-free (SPF) grade C57BL/6 male mice, 6 weeks old, were purchased from SLAC Laboratory Animal Co., Ltd. (China), and acclimatized for two weeks prior to the start of the experiment. The drinking water, straw mats, cages, and food used were autoclaved. All mice were housed in several cages, 4 or 5 per cage at 21°C, and fed a standard chow (SLAC Animal Laboratory). This experiment was approved by the Institutional Animal Care and Use Committee of SLAC (IACUC) Guide for Care and Use of Laboratory Animals (IACUC, No. 20190301t0180619).

### Mouse Model

**Fig. 1:**
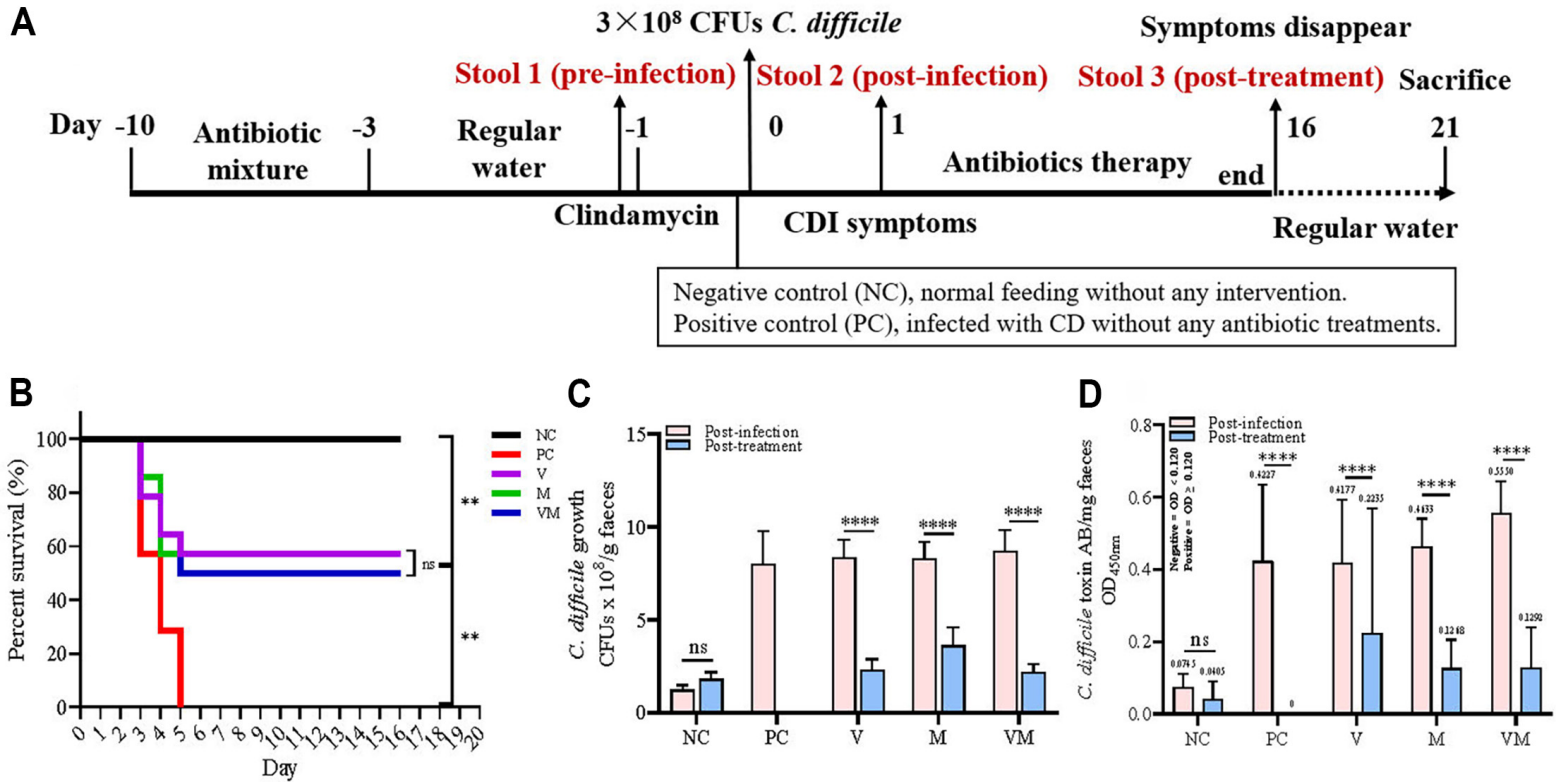
Animal assay. (**A**) Flow chart of the animal assay. CDI, *C. difficile* infection. CFUs, colony-forming units. Negative control (NC) mice were fed normally and without any intervention (except intraperitoneal injection of clindamycin). Positive control (PC) mice did not receive any antibiotic treatment after infection. (**B**) The final survival rate of mice. The Kaplan-Meier analysis was used for the survival curve. **, *p* < 0.01. ns, not significant. NC, negative control (*n* = 14). PC, positive control (*n* = 14). V, VAN (*n* = 14). M, MTR (*n* = 14). VM, VAN combined with MTR (*n* = 14). (**C**) Number of *C. difficile*. D. *C. difficile* toxin level. Level of toxin A/B presents in the feces of pCDI mice. OD_450nm_ < 0.12 represent negative, and OD_450nm_ ≥ 0.12 represent positive. The Dunnett’s multiple comparisons test was used for *C. difficile* colony count and level of toxin A/B. ns, not significant, ****p < 0.0001. The overall experiment was performed three times independently, with the number of mice being 4, 5, and 5 each time.

The pCDI mouse model was constructed using the previous method [16] (Fig. 1A). At the end of the acclimatization period, mice were randomly divided into five groups, each containing 14 mice. Except for the negative control (NC), all mice were continuously given drinking water containing a mixture of antibiotics (0.168 mg/ml colistin, 1.6 mg/ml kanamycin, 0.14 mg/ml gentamicin, 0.86 mg/ml metronidazole, and 0.18 mg/ml vancomycin) (Macklin, Shanghai, China) for 7 days, after which all mice received a single dose of clindamycin (10 mg/kg, Macklin) intraperitoneally. One day later, all mice (excluding NC) were gavaged with 3 × 10^8^ CFUs of *C. difficile* (CD). Antibiotic therapy was initiated after the onset of clinical signs (loose stools, rapid weight loss).

Negative control (NC) mice were fed normally and without any intervention (except intraperitoneal injection of clindamycin). Positive control (PC) mice did not receive any antibiotic treatment after CD infection. Based on the methodology of previous studies [13, 17], we slightly adjusted the dosage of antibiotics: V group (VAN, 50 mg/kg/day); M group (MTR, 50 mg/kg/day); VM group (co-administration of VAN and MTR, 50+50 mg/kg/day). The antibiotic solutions used for V (6.25 mg/ml), M (6.25 mg/ml), and VM (6.25+6.25 mg/ml) were prepared in advance and stored in a refrigerator at 4°C. In addition, 200 μl of the antibiotic solution was administered to each mouse by gavage every 12 h. The time point for treatment intervention is when mice first show significant weight loss, diarrhea, and other common clinical signs of pCDI. According to the method of Chen *et al*. [16], a variety of indicators such as diarrhea, body weight changes, and behavioral changes were combined to assess whether the mice reached a moribund state. Mice judged to be in a moribund state were euthanized. During the treatment stage, when the pCDI mice returned to normal and had no apparent clinical signs, the antibiotics were terminated, followed by normal feeding for a period of 5 days, and then all of the mice were euthanized.

### Histopathology

Except for the PC group, all mice had their cecum and colon tissues collected rapidly at the end of the experiment. In the PC group, as soon as the mice appeared to die, their intestinal tissues were collected for pathologic analysis. Cecum tissues were immersed in 4% paraformaldehyde and immobilized for 24 h at 4°C. After that, the tissues were stabilized with paraffin and cut into 5 μm sections using a microtome (Leica EM UC7, Leica, Germany). Finally, the sectioned tissue was stained with hematoxylin-eosin (H&E) and photographed under an Olympus microscope (Mod. U-LH100HG, Olympus, Japan).

### Fecal Sample Collection

Fresh fecal pellets were collected and rapidly placed in sterile Eppendorf (EP) tubes and stored at -80°C. A total of three sampling points were used. The first collection time point was within 24 h before the clindamycin injection. The second collection time point was at 24-36 h after infection, and the third collection time point was within 24-36 h after treatment cessation.

### Detection of *C. difficile* Numbers and Toxin Levels in Fecal Samples

The collected fecal samples were resuspended in sterile tubes containing PBS and homogenized. A portion of the fecal suspension was taken, diluted, and plated on *C. difficile* moxalactam norfloxacin agar (CDMN) (Oxoid, UK) for colony counting. A portion of the remaining fecal suspension was centrifuged and the supernatant was collected and assayed for toxin levels using the *C. difficile* TOXA/B II TM Kit (Tech Lab, USA).

### 16S rRNA Gene Amplicon Sequencing and Bioinformatic Analysis

Total fecal genomic DNA was extracted using an OMEGA DNA Kit (D5625-01) (Omega Bio-Tek, Norcross, USA) according to the manufacturer's instructions and stored at -20°C prior to further analysis. The quantity and quality of extracted DNA were individually determined by a NanoDrop ND-1000 spectrophotometer (Thermo Fisher Scientiﬁc, USA) and agarose gel electrophoresis. The forward primer 338F (5’-ACTCCTACGGGAGGCAGCA-3’) and the reverse primer 806R (5’-GGACTACHVGGGTWTCTAAT-3’) were used to amplify the V3-V4 region of the bacterial 16S rRNA gene. Sample-specific 7-bp barcodes were incorporated into the primers for multiplex sequencing. The PCR components consisted of 5 μl buffer (5×), 0.25 μl Fast Pfu DNA polymerase (5 U/μl) (Sangon Biotech, China), 2 μl dNTPs (2.5 mM), 1 μl each of forward and reverse primers (10 μM), 1 μl DNA template, and 14.75 μl ddH_2_O. The thermal cycle consisted of an initial denaturation at 98°C for 5 min, 25 cycles including denaturation at 98°C for 30 s, annealing at 53°C for 30 s, extension at 72°C for 45 s, and a final extension at 72°C for 5 min. PCR amplicons were purified with Vazyme VAHTSTM DNA cleaning beads (Vazyme, China) and quantified using a Quant-iT PicoGreen dsDNA Assay Kit (Invitrogen, USA). After the individual quantification steps, amplicons were pooled in equal amounts, and paired-end 2 × 300 bp sequencing was performed using the Illumina MiSeq platform with a MiSeq Reagent Kit v3 at Shanghai Personal Biotechnology Co., Ltd. (China).

The QIIME2 platform was used to perform microbial bioinformatics analysis according to the official tutorials [18]. The raw sequence data were demultiplexed using the demux plugin followed by primer cut using the cutadapt plugin [19]. Afterward, the sequences were then quality filtered, denoised, and merged, and chimeras were removed using the DADA2 plugin [20]. Nonsingleton amplicon sequence variants (ASVs) were aligned with mafft [21], and phylogenetic relationships were constructed using fasttree2 [22]. The ASV table in QIIME2 was used to calculate the alpha diversity index at the ASV level, with Observed species and Shannon as specific indications [23]. Beta diversity indices were estimated using the diversity plugin with a sequence sparsity of 18,607 per sample. Beta diversity analysis was performed using Bray-Curtis metrics and visualized by non-metric multidimensional scaling (NMDS) to investigate structural changes in microbial communities between samples [24]. Interactive presentation of microbial community taxonomic composition was carried out using Krona software (https://github.com/marbl/Krona/wiki) [25]. Principal component analysis (PCA) is based on the genus-level component profiles [26]. Linear discriminant analysis Effect Size (LEfSe) analysis was conducted using Galaxy platform (http://huttenhower.sph.harvard.edu/galaxy/) [27]. Prediction of microbial function was performed using the phylogenetic investigation of communities by reconstruction of unobserved states (PICRUSt2) based on the MetaCyc database (https://metacyc.org/), and KEGG database (https://www.kegg.jp/) [28].

### Untargeted Metabolomics

First, fecal pellets (100 mg) were added to an Eppendorf (EP) tube containing 2-chlorophenylalanine methanol (-20°C, 4 ppm, 0.6 ml) with a 30 s vortex oscillation. Second, 100 mg of glass beads were added and further ground for 90 s (60 Hz) using a high-throughput tissue grinder and sonicated for 10 min at room temperature. Third, samples were centrifuged to collect the supernatant (0.22 μm, sterile filter) for LC-MS (UltiMate 3000-Q Exactive Focus, Thermo Fisher Scientific), and 55 μl of each sample supernatant was mixed into quality control (QC) samples [29, 30].

Chromatographic separations were performed in a Thermo Ultimate 3000 system equipped with an ACQUITY UPLC HSS T3 (150 × 2.1 mm, 1.8 μm, Waters) column (Thermo Fisher Scientiﬁc) maintained at 40°C. The autosampler temperature was set to 8°C. The analytes were eluted with a gradient of 0.1% formic acid aqueous solution (C) and 0.1% formic acid acetonitrile solution (D) or 5 mM ammonium formate aqueous solution (A) and acetonitrile solution (B) at a flow rate of 0.25 ml/min. After equilibration, 2 μl of each sample was injected. The linear gradient of solvent B (v/v) was: 0-1 min, 2% B/D; 1-9 min, 2-50% B/D; 9-12 min, 50-98% B/D; 12-13.5 min, 98% B/D; 13.5-14 min, 98-2% B/D; 14-20 min, 2% D-positive model (14-17 min, 2% B-negative model). The ESI-MSn experiments were performed on a Thermo Fisher Q Exactive Focus mass spectrometer with the spray voltages of 3.8 kV and -2.5 kV in positive and negative modes, respectively. The sheath gas and auxiliary gas were set to 30 and 10 arbitrary units, respectively. The capillary temperature was 325°C. The analyzer scanned over a mass range of m/z 81-1 000 for a mass resolution of 70,000. Data-dependent acquisition (DDA) MS/MS experiments were performed using HCD scanning. The normalized collision energy was 30 eV. A dynamic exclusion method was used to remove some unnecessary information from the MS/MS spectra [31]. Based on the base peak chromatography (BPC), quality control (QC), and quality assurance (QA), it was determined that the QC sample dense distribution data were reliable. The QC samples were collected with good reproducibility, indicating that the system was stable. In the QC samples, the characteristic peak ratio of RSD (<30%) reached approximately 70%, indicating positive data. In addition, we performed differential metabolic pathways and metabolites analysis.

### Statistical Analysis

The obtained data in this study were performed using Minitab Statistical Software (version 20) (Minitab Inc., USA). One-way ANOVA and two-tailed *t*-tests were used to analyze the statistical significance of the data. The Kaplan-Meier analysis was used for the survival curve. The alpha diversity index label is the *p*-values obtained from the Kruskal-Wallis test. A *p*-value of < 0.05 was considered statistically significant.

## Results

### Survival Rate and Change of Fecal Microbiota in Mice

**Fig. 2:**
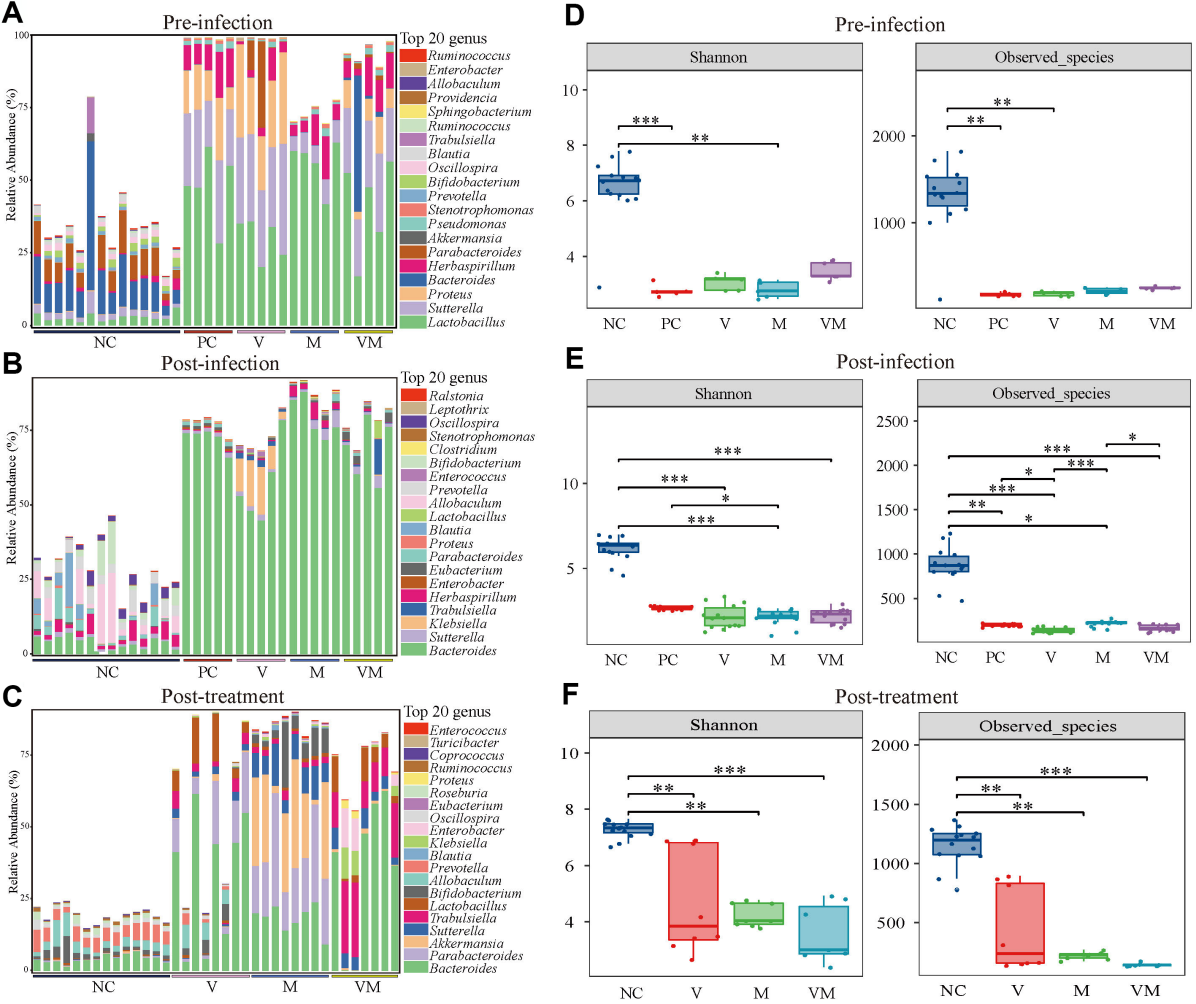
Fecal microbial composition. Top 20 members in relative abundance at the genus level at the pre-infection (**A**) the post-infection (**B**) and the post-treatment stages (**C**). Alpha diversity levels at the pre-infection (**D**), the post-infection (**E**), and the post-treatment stages (**F**). Shannon and Observed species indices were used to reflect the level of Alpha diversity. The horizontal coordinates are the grouping labels and the vertical coordinates are the values of the corresponding alpha diversity indices. In the box-and-line plot, the meanings of the symbols are as follows: upper and lower end lines of the box, upper and lower quartiles (Interquartile range (IQR)); median line, median; upper and lower margins, maximum and minimum inner circumference values (1.5 times IQR); and points outside the upper and lower margins, indicating outliers. The Kruskal-Wallis test was used for alpha diversity. ***p < 0.001, **p < 0.01, *p < 0.05.

The final survival rate of each group was NC (100%) > V (57%) = M (57%) > VM (50%) > PC (0%) in descending order (Fig. 1B), and there was no significant difference between V, M, and VM. The number of *C. difficile* and toxin production in the stool increased rapidly during the infection stage, and this trend decreased after treatment, but the toxin test remained positive at the endpoint (Fig. 1C and 1D). As seen in the intestinal morphology and cecum-stained sections, compared to the negative control, the intestinal tissue of mice that received antibiotics treatment was severely hyperplastic and edematous, especially in V and VM (Fig. S1A). Analysis of the composition of the fecal microbiota revealed that continuous intake of mixed antibiotics prior to infection resulted in a rapid increase in *Proteus*, *Sutterella*, and *Lactobacillus*, with a combined relative abundance approaching 75% (Fig. 2A), which was accompanied by a significant downregulation of Shannon index and Observed species levels (Fig. 2D). With the invasion and infection of *C. difficile*, the relative abundance of *Bacteroides* gradually dominated absolutely (Fig. 2B), while the Shannon index and Observed species levels remained lower in these infected groups than in the negative control during the same period (Fig. 2E). At the post-treatment stage, the abundance of *Bacteroides* decreased but still occupied about 50%. Meanwhile, the abundance of *Parabacteroides* and *Lactobacillus* increased in the V group, as did *Parabacteroides*, *Akkermansia*, *Bifidobacterium*, *Sutterella* in the M group and *Trabulsiella*, *Lactobacillus*, *Enterobacter*, *Klebsiella* in the VM group (Fig. 2C). However, there was still no significant fluctuation in the level of α-diversity, and V, M, VM were basically the same and significantly lower than NC (Fig. 2F). Further assessment of β-diversity revealed that except for NC, where samples were highly aggregated, and PC and M, where samples were highly dispersed, sample dispersion was insignificant for both V and VM (Fig. S1B).

### Differences in Microbial Members between Groups and Identification of Microbial Biomarkers

**Fig. 3:**
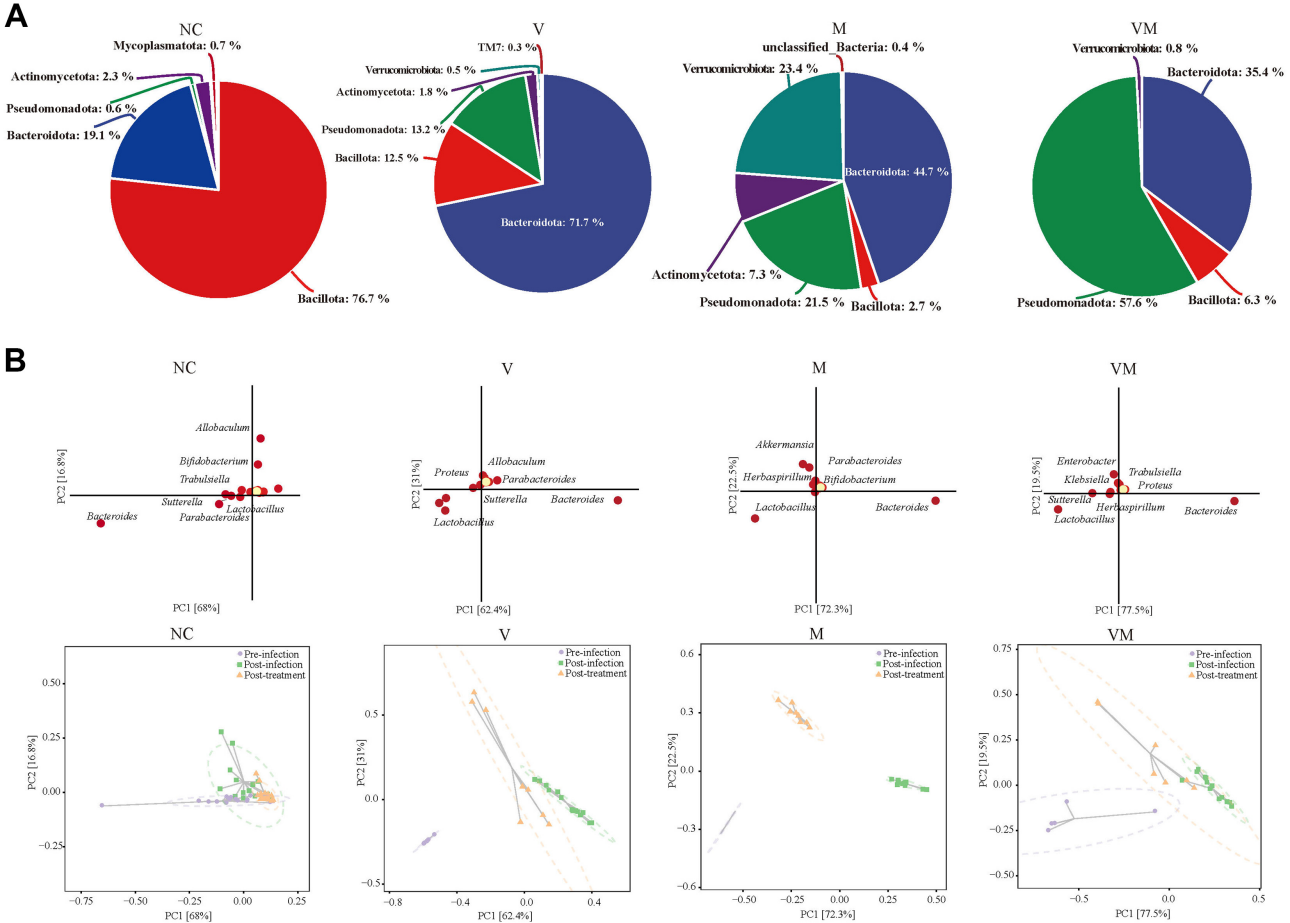
Microbial member differences between groups and identification of microbial biomarker. (**A**) Krona taxonomic map of phylum-level microbes at the post-treatment stage. (**B**) PCA loadings plot and scores plot of genus-level microbes. Each point of the figure represents a genus-level microbe, and the horizontal and vertical coordinates of the point can be thought of as the magnitude of the microbe contribution to the samplés variance in these two dimensions, respectively. Percentages in parentheses on each of the two axes are the ratio of the difference in species abundance composition to the total difference for all samples in that dimension. The ratio of physical unit lengths of the two axes is set by default to be the same as their explanatory ratio, so that the contribution of a microbe to the difference in composition between sample groups is proportional to the sum of its distances to the axes, and is indicated by a color from yellow to red indicating its value from small to large. Each dot in the scores plot represents a sample, with different colored dots indicating different samples (groups).

Based on the results of the differences in microbial community composition (β-diversity), we further explored which microbe differential distributions were mainly responsible for these differences. As shown in the Krona microbial composition diagram, after treatment, the relative abundance of microbes (phylum level) in NC was higher in *Bacillota* (76.7%) and *Bacteroidota* (19.1%); the relative abundance of microbes in V was higher in *Bacteroidota* (71.7%), *Pseudomonadota* (13.2%), and *Bacillota* (12.5%); the relative abundance of microbes in M was higher in *Bacteroidota* (44.7%), *Verrucomicrobia* (23.4%), and *Pseudomonadota* (21.5%), and in VM the relative abundance of microbes was higher in *Pseudomonadota* (57.6%), *Bacteroidota* (35.4%), and *Bacillota* (6.3%) (Fig. 3A). It was worth noting that the share of *Verrucomicrobia* in V, M, and VM was 0.5, 23.4, and 0.8%, respectively. Genus-level microbes were analyzed and presented in PCA plots by dimensionality reduction. *Bacteroides* and *Lactobacillus* were shared contributing microbes of V, M, and VM (Fig. 3B). The characteristic contributing microbes were different among the groups, such as *Sutterella* and *Allobaculum* in V, *Akkermansia* and *Bifidobacterium* in M, and *Enterobacter*, *Klebsiella*, and *Trabulsiella* in VM. We further used LDA Effect Size (LEfSe) analysis to analyze differences at the taxonomic level for all microbial members simultaneously to find robustly differential microbes between groupings, and the results were also cross-checked with those obtained by previous microbial taxonomic analysis methods. At the post-treatment stage, the genus-level microbial biomarkers in V were *Bacteroides* and unidentified_S24_7, and in M they were *Akkermansia* and *Bifidobacterium*, while in VM they were *Enterobacteriaceae*, *Klebsiella*, and *Trabulsiella* (Fig. S1C). The above three analysis methods showed that the microbial biomarkers after V, M, and VM treatment are different.

### Correlation Analysis of Fecal Microbiota and Metabolome

**Fig. 4:**
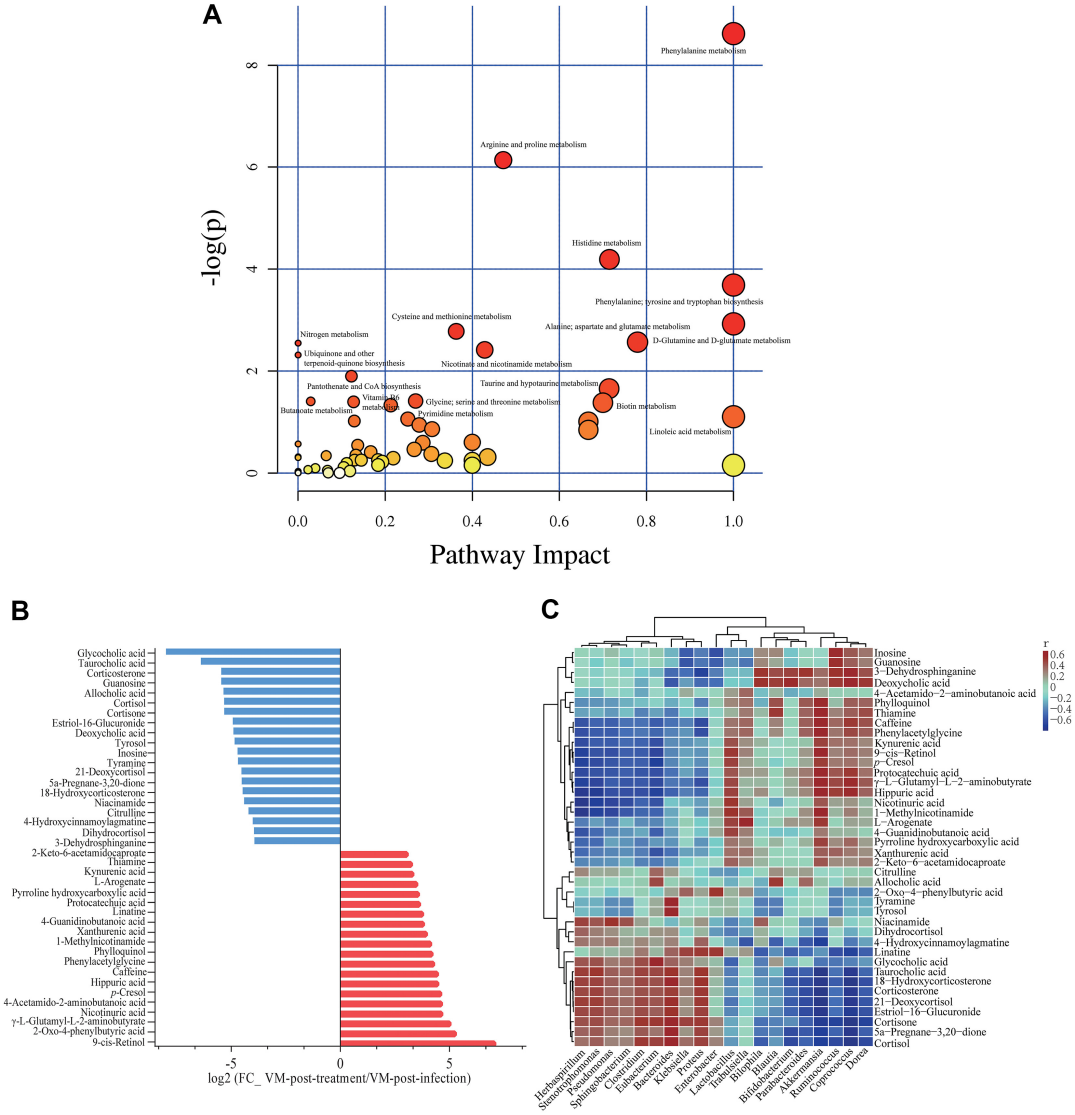
Correlation analysis of differential metabolite and microbiota data after VM treatment. (**A**) Map of factors affecting differential metabolic pathways after VM treatment. (**B**) The top 20 metabolites changed most significantly before and after VM treatment. (**C**) Heatmap of correlations between genus-level microbial abundance and metabolome data. Calculation of the Bray-Curtis distance matrix for the two data sets ‘metabolome’ and ‘microbial composition’ utilizing the R package ‘vegan’, followed by the Mantel test statistical test utilizing the QIIME2 software and the permutation test for the samples (999 times). The statistical significance of the similarity between the metabolomics data and the microbial composition data was assessed (*p*-value < 0.05) and a *p*-value = 0.001 was determined, which indicates significance. Using Mothur software, Spearman rank correlation coefficients were calculated between metabolomics data and microbial abundance, and heatmaps were plotted based on the results of the correlation coefficient matrix (rho correlation coefficients are values between -0.6 and 0.6; when -0.6<rho<0, the two are negatively correlated; when 0<rho<0.6, the two are positively correlated; and when rho=0, the two are not correlated). If the correlation between the two is positive, it will be shown in red, and vice versa, if it is negative, it will be shown in blue; the color indicates the strength of the correlation.

The software PICRUSt2 is a tool that predicts the functional abundance of a sample based on the abundance of marker genes it contains. Using the reference genomic data that comes with the software, functional predictions can be made for 16S rRNA sequences. PICRUSt2 is capable of predicting 16S rRNA gene sequences in several functional databases, including MetaCyc (https://metacyc.org/), and KEGG (https://www.kegg.jp/), etc. The core of the KEGG database is the KEGG pathway database (http://www.genome.jp/kegg/pathway.html), which categorizes metabolic pathways into six major groups, including metabolism, and genetic information processing, environmental information processing, cellular processes, organismal systems, and human diseases (Fig. S2A). We focused on the abundance of the predicted KEGG secondary function pathways in the V, M, and VM groups and found that the highest abundance of pathways was under metabolism, mainly in carbohydrate metabolism, amino acid metabolism, and metabolism of cofactors and vitamins (Fig. S2A). Pathways related to cellular processes, environmental information processing, and genetic information processing ranked second, third, and fourth, respectively, and there were no notable differences between the groups. In the MetaCyc pathway prediction, the main metabolic pathways of V, M, and VM are focused on biosynthesis, including metabolic sub-pathways such as amino acid, cofactor, vitamin, fatty acid, lipid, and carbohydrate biosynthesis (Fig. S2B). Pathways related to the degradation/utilization/assimilation and generation of precursor metabolites and energy ranked second and third, respectively, and there were no notable differences between the groups. A combination of the predictions from the two databases showed that the metabolic activity of the gut microbiota under the V, M, and VM treatments was mainly focused on amino acid and carbohydrate utilization. We further analyzed mouse feces using non-targeted metabolomics techniques and correlated metabolomic data with microbiota data. The amino acid pathways represented by the metabolism of phenylalanine, arginine, proline, and histidine were highly enriched under the co-administration treatment (Fig. 4A), which is consistent with the PICRUSt2 prediction that amino acid metabolism is the most variable metabolic pathway in VM. The levels of 9-cis-retinol, γ-L-glutamyl-L-2-aminobutyrate, nicotinuric acid, *p*-cresol, caffeine, and 2-Oxo-4-phenylbutyric acid were significantly increased in the feces of VM-treated mice, while the levels of primary bile acids glycocholic acid and taurocholic acid, secondary bile acid deoxycholic acid (DCA), and corticosterone levels were significantly decreased (Fig. 4B). The results of correlation analysis showed that glycocholic acid, taurocholic acid, and corticosterone were highly positively correlated with genus-level microbes *Eubacterium*, *Bacteroides*, *Klebsiella*, *Proteus*, *Pseudomonas*, and DCA was highly positively correlated with *Blautia*, *Bifidobacterium*, *Parabacteroides*, *Akkermansia*, *Ruminococcus*, *Coprococcus*, and *Dorea*, while the top five metabolites that significantly increased were positively correlated with *Akkermansia*, *Lactobacillus*, *Enterobacter*, and *Klebsiella* (Fig. 4C).

## Discussion

The co-administration of antibiotics is not uncommon in many clinical disease treatments, and its main purpose is to improve drug efficacy, reduce drug toxicity, and prevent the development and evolution of antibiotic resistance, etc. [32]. However, the co-administration of VAN and MTR was applied in some cases of clinical CDI treatment, but the reasons for the wide variation in efficacy are unknown. Such a pattern of antibiotic combinations is more often than not determined by physician experience. Here, we found that the final survival rates of pCDI mice treated with VAN and MTR alone or in combination were similar, which is consistent with some of the known clinical results [5].

CDI as a bacterial intestinal infection, and its occurrence, development and treatment are centered on the intestinal ecology, in which the gut microbiota and metabolites play a decisive role [33]. At the pre-infection stage, we found that the continuous intake of mixed antibiotics prior to infection led to a significant change in the structure of the normal mouse microbiota, with a rapid decrease in the number of microbial members and the level of alpha diversity. The abundance especially of *Oscillospira*, *Bifidobacterium*, and *Blautia*, which are associated with short-chain fatty acids (SCFAs) production, was rapidly downregulated [34], while *Lactobacillus*, *Sutterella*, *Proteus*, *Herbaspirillum* and *Pseudomonas* rapidly increased, with an abundance percentage close to 100% (Fig. 2). *Lactobacillus* is a group of antibiotic-resistant microbes in the gut microbiota, so the antibiotic environment contributes to the colonization and expansion of *Lactobacillus* [35]. It should be seen that even though *Lactobacillus* accounts for more than 50%, the percentage of *Sutterella* and *Proteus* that accompany its rise is likewise close to 50%, and yet this trend does not appear in the M group. The mixed antibiotic-treated mouse microbiota did not differ significantly between groups at the order or family classification levels (data not shown). In fact, the relative abundances of some microbes are not directly related to whether they are core gut members that regulate and influence the functioning of the entire microbiota, and thus further validation is needed through a variety of analytical tools. This microbial composition at this stage indicated that the overall number of microbes was reduced in the antibiotic environment, the number and abundance of harmful members were higher, and the ecological balance had been disrupted. At the post-infection stage, this trend was further intensified after the invasion of *C. difficile*, where the dominant members *Lactobacillus*, *Parabacteroides*, and *Herbaspirillum* were replaced by *Bacteroides* and *Klebsiella*. At the post-treatment stage, the microbial structure in the co-administration group was close to that at the post-infection stage, which means that co-administration did not significantly alter the existing microbiota, whereas *Verrucomicrobia* appeared in both the antibiotic mono- or co-administration groups during this period, and this species was more abundant in M than in V and VM. *Akkermansia* is known to be the only genus belonging to *Verrucomicrobia*, so the increase or decrease in *Verrucomicrobia* abundance can also be indicated by *Akkermansia* abundance [36]. *Akkermansia* has been shown to exhibit positive effects in host metabolic regulation, immune homeostasis, and inflammation mitigation [36]. At the end of the treatment, the high abundance of *Akkermansia* meant that the inflammation from the infection was greatly relieved. *Pseudomonadota*, another highly abundant species at the phylum level, accounts for a relatively high proportion of the gut microbiota and contains a variety of harmful microbes, such as *Escherichia coli*, *Salmonella*, *Vibrio cholera*, and *Helicobacter pylori* [37]. At the treatment stage, co-administration prompted *Pseudomonadota* to account for 57.6%, far more than the levels seen in VAN or MTR monotherapy. Meanwhile, among *Pseudomonadota*, the genus-level members *Enterobacter*, *Klebsiella*, and *Shigella* were the main contributors, and these results indicated that the co-administration substantially downregulated *Verrucomicrobia* abundance and significantly upregulated *Pseudomonadota* abundance. In addition, microbial biomarkers appearing under VAN monotherapy are unidentified_S24-7, which has been shown to negatively correlate with pro-inflammatory cytokines in the mouse intestine and to alleviate experimental colitis [38]. A noteworthy point is that *Akkermansia* and *Bifidobacterium* dominated the changes in the microbiota induced by MTR monotherapy. In terms of the composition of the normal gut microbiota, the efficacy of MTR was superior to that of VAN and their co-administration in this study. Although MTR is no longer recommended for CDI treatment in Europe or the U.S., more consideration should be given to individual symptoms, regions, and even ethnicity in the actual diagnosis and treatment of CDI [9].

The activity of amino acid metabolic pathways represented by phenylalanine, arginine, tyrosine, proline, histidine, and ornithine metabolism was substantially upregulated after treatment. These amino acids are involved in the Stickland reaction of *C. difficile*, producing ATP and NAD+, and play an important role in *C. difficile* colonization and infection [39]. The levels of 9-cis-retinol, 2-Oxo-4-phenylbutyric acid, γ-L-glutamyl-L-2-aminobutyrate, and nicotinuric acid increased significantly after co-administration treatment and were accompanied by an increase in the abundance of specific microbes. Meanwhile, the levels of bile acids (BAs) decreased significantly. The metabolism of primary bile acids promotes *C. difficile* spore germination and accelerates the infection process, while secondary bile acids have the opposite effect [40]. Co-administration reduced the DCA and its associated microbes *Bifidobacterium*, *Parabacteroides*, and *Akkermansia*. Notably, the microbes that were associated with increased or decreased metabolites in the feces at the end of co-administration treatment were not all ‘good microbes’ or ‘bad microbes’ in the traditional sense. Both appeared to coexist, implying that co-administration treatment indiscriminately weakened or increased the microbial number and relative abundance of antibiotic-sensitive microbes, including both beneficial and harmful members. In this study, co-administration was moderately effective in the treatment of pCDI mice and was accompanied by severe intestinal hyperplasia and edema. Animal models are one of the most important tools for investigating human diseases. Hamsters have been used in the construction of CDI models; however, compared to the slow onset of clinical CDI patients, the hamster model is accompanied by rapid onset and death and is therefore not suitable as a CDI model [41]. In contrast, C57BL/6 mice exhibit a relatively slow onset of CDI, which is more consistent with the human CDI profile. Chen *et al*. successfully constructed a now widely recognized CDI mouse model approaching the human CDI profile by disrupting the normal microbiota structure of conventional C57BL/6 mice with a mixture of antibiotics, reducing colonization resistance, and facilitating toxin production and infection [16]. However, it must be understood that their model, although capable of simulating the CDI pathogenesis process, is still far from the actual clinical CDI development and drug treatment model, and therefore, it is more appropriate as a preclinical investigation.

In conclusion, the co-administration of VAN and MTR did not alter the survival rate of pCDI mice compared to VAN or MTR monotherapy. Co-administration enhanced the activity of amino acid metabolic pathways represented by phenylalanine, arginine, proline, and histidine, decreased the level of secondary bile acids represented by DCA, and downregulated the abundance of beneficial microbes, such as *Bifidobacterium* and *Akkermansia*. At the same time, the antibiotic-sensitive microbial members and their relative abundance, including beneficial and harmful microbes, were reduced or increased indiscriminately. Taken together, there may be potential risks associated with the combination of VAN and MTR in the treatment of CDI.

## Supplemental Materials

Supplementary data for this paper are available on-line only at http://jmb.or.kr.
