# Supplementary material for: Efficacy Assessment of the Co-Administration of Vancomycin and Metronidazole in Clostridioides difficile-Infected Mice Based on Changes in Intestinal Ecology
Source: J Microbiol Biotechnol. 2024 Feb 29;34(4):828–37. doi: 10.4014/jmb.2312.12034 (PMC11091681; doi:10.4014/jmb.2312.12034)
Supplement: Supplementary file 1 [file jmb-34-4-828-supple.pdf]

J. Microbiol. Biotechnol.
https://doi.org/10.4014/jmb.2312.12034

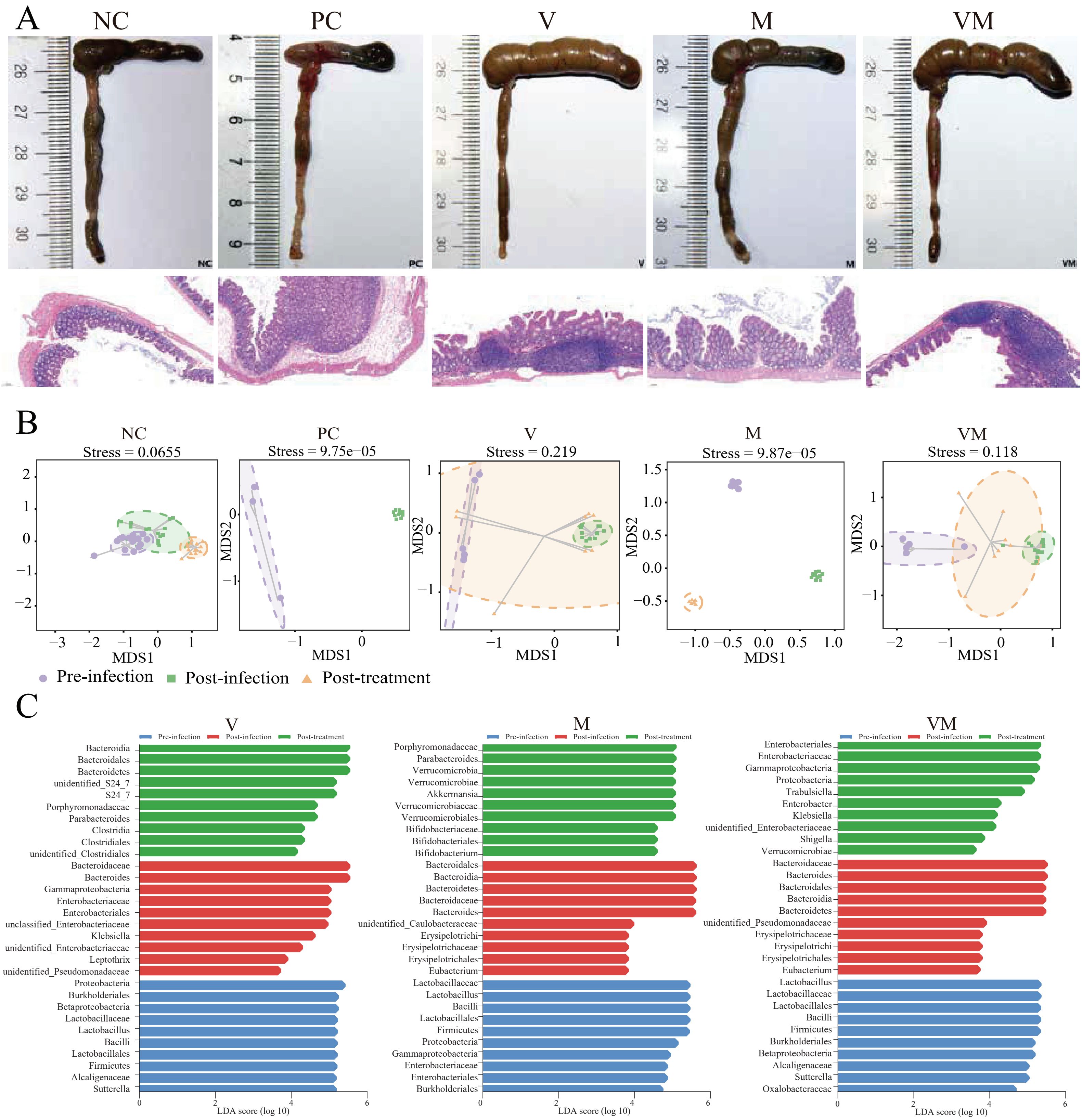

Fig. S1. A. Mouse intestinal histomorphology and cecum H&E-stained sections. B. Nonmetric multidimensional scaling (NMDS). C. LEfSe analysis. The classification units are ranked according to the magnitude of the score value, which describes their specificity in the sample grouping.

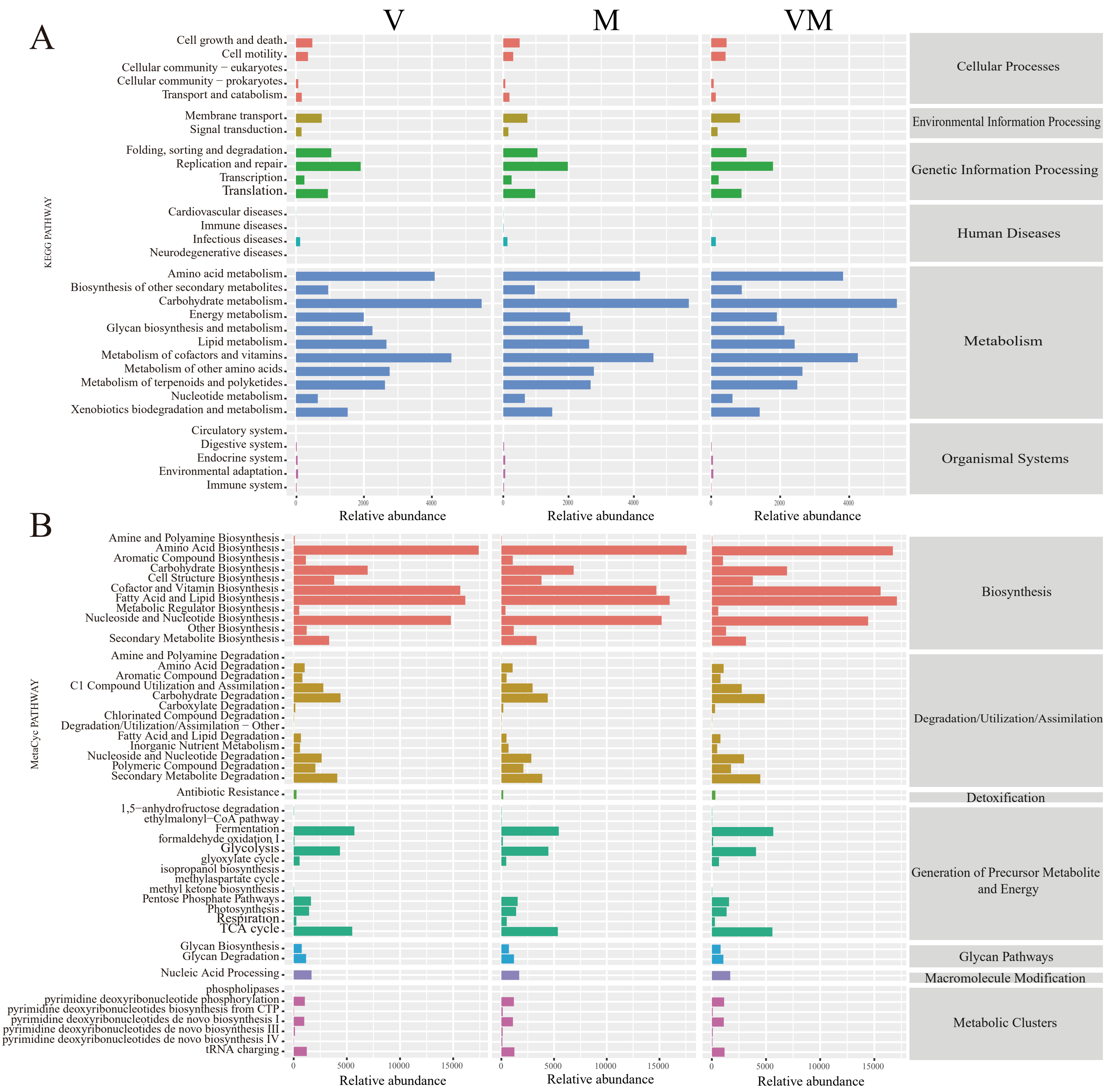

Fig. S2. Relative abundance maps of predicted KEGG and MetaCyc pathways in V, M, and VM. A. Predicted KEGG secondary functional pathway abundance map. B. Predicted abundance map of MetaCyc secondary functional pathways.
